# Natural Compounds as Regulators of the Cancer Cell Metabolism

**DOI:** 10.1155/2013/639401

**Published:** 2013-05-16

**Authors:** Claudia Cerella, Flavia Radogna, Mario Dicato, Marc Diederich

**Affiliations:** ^1^Laboratoire de Biologie Moléculaire et Cellulaire du Cancer, Hôpital Kirchberg 9, Rue Edward Steichen, 2540 Luxembourg, Luxembourg; ^2^Department of Pharmacy, College of Pharmacy, Seoul National University, Seoul 151-742, Republic of Korea

## Abstract

Even though altered metabolism is an “old” physiological mechanism, only recently its targeting became a therapeutically interesting strategy and by now it is considered an emerging hallmark of cancer. Nevertheless, a very poor number of compounds are under investigation as potential modulators of cell metabolism. Candidate agents should display selectivity of action towards cancer cells without side effects. This ideal favorable profile would perfectly overlap the requisites of new anticancer therapies and chemopreventive strategies as well. Nature represents a still largely unexplored source of bioactive molecules with a therapeutic potential. Many of these compounds have already been characterized for their multiple anticancer activities. Many of them are absorbed with the diet and therefore possess a known profile in terms of tolerability and bioavailability compared to newly synthetized chemical compounds. The discovery of important cross-talks between mediators of the most therapeutically targeted aberrancies in cancer (i.e., cell proliferation, survival, and migration) and the metabolic machinery allows to predict the possibility that many anticancer activities ascribed to a number of natural compounds may be due, in part, to their ability of modulating metabolic pathways. In this review, we attempt an overview of what is currently known about the potential of natural compounds as modulators of cancer cell metabolism.

## 1. (Re-)Evaluating the Targeting of Metabolic Alterations in Cancer

Deregulated metabolism is one of the oldest mechanisms associated with cancer physiology. The actual meaning and the selective advantages induced by this deregulation remain nowadays still a matter of debate despite the pioneering work of Warburg about the impact of the alteration of the energetic metabolism in cancer cells. Certainly, several reasons have significantly contributed to delay the advancement in this area of investigation. For many years, the search for new anticancer therapeutic agents has been extremely focused on fighting the two most intuitive altered features of cancer cells, namely, their sustained and uncontrolled proliferation and their ability of evading death. Accordingly, we have assisted over the years in the development of different classes of therapeutic agents reducing cancer cell proliferation or inducing cancer cell death. The main target of these studies was the differential susceptibility of cancer versus normal cells to these treatments. Over the time, however, we have also learned about the limits of this approach considering the high incidence of therapeutic failure and the frequent development of systemic toxicity.

Recently, the high level of complexity and heterogeneity of cancer allowed considering this disease as a dynamic multicellular system with complex forms of interactions and cellular communications with the own environment. It has become evident that consolidated cancer hallmarks including sustained and uncontrolled cell proliferation and resistance to cell death need to be reconsidered in a much more complex modulatory context if we want to therapeutically succeed with cancer.

At the light of this new vision, the ability of cancer cells to reprogram their cellular energetic metabolism is passing through a renaissance of interest in cancer biology for these chapters of fundamental biochemistry. The discovery of unexpected cross-talks between well-known metabolic factors and mediators of unrelated processes is fuelling this renewed interest. On one side, noncanonical regulatory functions of specific metabolic enzymes or substrates are emerging; on the other side, oncogenes, tumour suppressors, as well as modulators controlling events typically altered at the very early stages of cancer progression including immune response, cell proliferation, or cell death appear in the dual role of controlled/controllers of metabolic processes. Decoding the roles of metabolic changes occurring during carcinogenesis and identifying the key nodes that differentiate pathological and healthy behavior have two important implications: novel predictive biomarkers and new drug discovery strategies. Consequently, additional knowledge may offer new tools to troubleshoot frequent chemotherapeutic failures; additionally, compounds targeting metabolic processes may also be potentially used for chemopreventive purposes. This research is only emerging, transforming the identification of metabolically active agents into an opportune challenge.

Nature provides a considerable source of biologically active compounds with a diversified pharmacological potential. Remarkably, almost 80% of all anticancer compounds are isolated from plants, fungi, and microorganisms. Both natural and chemically modified molecules (in order to improve stability, specificity, and/or activity) are able to counteract each of the cancer hallmarks [1, 2] recently reclassified by Hanahan and Weinberg [3]. Accumulating evidence also concerns cancer metabolism [2]. Remarkably, many of these compounds are food constituents or have been used since a long time in traditional medicine. Thus, they show a favorable profile in terms of their absorption/metabolism in the body with low toxicity.

## 2. Advantages of Altered Metabolism in Cancer versus Normal Cells

### 2.1. Metabolic Switch from Mitochondrial Respiration to Glycolysis

The preferential switch from oxidative phosphorylation to aerobic glycolysis represents the most discussed and investigated altered metabolic feature of cancer cells and was first described by Otto Warburg in the 1920s. He already hypothesized mitochondrial dysfunctions as the causative event. Defects in the enzymatic respiratory chain exist in cancer cells [4]; however, there is no clear correlation between the incidence of mitochondrial dysfunctions and the metabolic switch to glycolysis, the latter being commonly reported in cancer cells. In a number of instances, instead, cancer tissues/cells even consistently rely on mitochondrial respiration to produce ATP [5]. Furthermore, under specific circumstances, cancer cells may also be forced to reactivate mitochondrial energy production [6]. These observations clearly show that mitochondria are generally functional in cancer cells and support the hypothesis that the propensity of cancer cells to exacerbate the glycolytic pathway, while decreasing oxidative phosphorylation, must be an active option conferring important advantages despite the evident energetic inefficiency of glycolysis. Nevertheless, identification of these selective advantages is not an obvious task, being indeed matter of debate. 

Theoretically, metabolic alterations during carcinogenesis could provide multiple benefits as cancer cells need to satisfy a continuous demand in macromolecule precursors to maintain their high proliferation rate. As a matter of fact, the reduction of mitochondrial respiration prevents a complete degradation of glucose to carbon dioxide (CO_2_) and water and leads to accumulation of precursors used by the major cellular synthesis pathways leading to amino acids, nucleotides, and lipids. Consequently, this metabolic alteration inevitably fuels these anabolic pathways. Second, cancer cells experience moderate to severely reduced oxygen tension, and the fact to preferentially exploit glycolysis to produce energy in this situation represents an interesting adaptation. Accordingly, overexpression or stabilization of the hypoxia-inducible factor (HIF) in response to low-oxygen conditions promotes the glycolytic metabolism, by inducing transcription of glucose transporters and numerous key glycolytic enzymes [7].

An increased glycolytic flux means also very frequently overexpression and/or increased activity of specific isoforms of several glycolysis-related enzymes. Glucose transporters, or key enzymes as hexokinase II (HKII), glyceraldehyde-3-phosphate dehydrogenase (GAPDH), lactate dehydrogenase (LDH) and the isoform M2 of pyruvate kinase (PKM2) are upregulated in cancer cells and accordingly suggested as potential therapeutic targets [8, 9]. Interestingly, nonglycolytic functions are also emerging for several of these enzymes, and the novel activities ascribed do further promote cancer aggressiveness. For example, GAPDH, LDH, or PKM2 may additionally activate gene expression by working as direct transcriptional factors or by interacting with and, thereby, modulating the activity of other nuclear proteins [10–12] (including HIF-1 and the Signal Transducer and Activator of Transcription 3 (STAT3) [13, 14]) required in the transcription of genes especially implicated in cell proliferation (e.g., histones H2A and H2B, MEK5, c-Myc, cyclin D1, and androgen receptor [10, 13–15]).

The hyperproduction of lactate plays a dual role. On one side, it activates the glycolytic pathway, ensuring the regeneration of nicotinamide adenosine diphosphate (NAD^+^), as part of a feedback regulatory mechanism; on the other side, it is secreted outside the cells where it promotes angiogenesis and spreading of cancer cells from their primary site. A mutual control exists between events controlling lactate production and synthesis of proangiogenic factors. For example, the extracellular acidification due to the transport of lactate coupled to H^+^ extrusion promotes upregulation of HIF-1 [16, 17]. HIF-1, in turn, transactivates the LDH-A promoter [16]. Besides, acidic conditions destabilizes the behavior of the immune system, which further contributes to cancer invasion. Lactate secretion, indeed, impairs the function of specific immune cells (including cytotoxic T lymphocytes) and cytokine production [18]. Furthermore, it promotes cell motility by controlling the expression level of constituents of the matrix [19, 20]. 

Identifying further advantages of the Warburg effect, other intriguing explanations involve mitochondria. Reducing the mitochondrial metabolism may inevitably decrease accumulation of reactive oxygen species (ROS). Suppression of ROS formation has been suggested as an important advantage for rapidly proliferating cell systems; these cells may be better protected against the risk of DNA damage during DNA synthesis [21]. This model seems to be encouraged by the observation that healthy highly proliferating systems temporarily switch to glycolysis before entering in S-phase [22]. Moreover, c-Myc, which activates transcription of the glycolytic enzymes HKII, enolase-1 (ENO-1), and LDH (subunit A), further promotes this switch in concomitance with the entry in S-phase [22–24].

More recently, unconventional roles were ascribed to pyruvate, the end product of glycolysis, which is massively converted into lactate in cancer cells instead of being transported into mitochondria to initiate mitochondrial metabolism. The plasma membrane transporter SLC5A8 was reported to be downregulated in different human cancer cells [25, 26]. Its silencing occurs at very early stages of carcinogenesis; moreover, the restoration of its expression triggers cell death [27]. Accordingly, it has been hypothesized that SLC5A8 may act as a tumour suppressor. This transporter couples Na^+^ extrusion to the intake of extracellular monocarboxylates, including pyruvate, into the cell. The group of Ganapathy has proposed an interesting model to explain the tumour suppressor activity of SLC5A8 specifically centered on the role of pyruvate [28]. According to their findings, pyruvate acts as a specific inhibitor of histone deacetylase-(HDAC-)1 and -3 isoforms, an event that in turn promotes cell death [28]. Therefore, keeping low levels of pyruvate may stabilize specific epigenetic aberrations established in cancer cells and promote cancer cell survival. Remarkably, pyruvate is maintained at very low levels in cancer cells [27]. Accordingly several mechanisms may participate in buffering the intracellular pyruvate levels together with upregulated LDH-A in cancer cells. They include also transporters as SLC5A8 and, conceivably, other monocarboxylate-specific transporters whose expression is modulated in cancer cells [29]. This model has fascinating implications. It assigns to pyruvate itself the role of a tumour suppressor [27]. Therefore, the control of intracellular pyruvate levels could play an active and central role in the altered metabolic profile of cancer cells. Moreover, it prompts us to consider additional roles for typical altered metabolic conditions in cancer cells that deal directly or indirectly with pyruvate accumulation. The preferential expression of the less efficient dimeric form of PKM2 (slowly accumulating pyruvate) or the relevance of the exacerbated conversion of pyruvate into lactate would be two interesting conditions to further investigate. In addition, these considerations remind us how much each metabolic alteration in cancer may play multiple functions, well exploited by cancer cells to succeed and ultimately survive and proliferate.

### 2.2. Relevance of Other Altered Metabolic Pathways in Cancer

Preferential exploitation of aerobic glycolysis by cancer cells is a key issue of reprogrammed metabolism. It is becoming clear that other metabolic pathways or mediators may play a fundamental role in cancer. The availability of recent sophisticated experimental approaches to study the metabolic profile of cancer cells has allowed identification of an impressive number of alterations. They essentially concern levels of expression/accumulation or status of enzymes or intermediate substrates involved in several anabolic pathways. Despite the evident advantage of these modifications within the anabolic process in which they are mainly involved, additional noncanonical functions have emerged, including control of redox homeostasis or specific signalling events enabling the high cellular proliferation rate. In this section, we will briefly discuss two key pathways suitable for therapeutic targeting.

#### 2.2.1. Glutamine Metabolism

Beside glucose, cancer cells frequently rely on glutamine metabolism. This amino acid is uptaken through specific transporters and directed to the mitochondria where it is converted first in glutamate (by a mitochondrial glutaminase). Glutamate then fuels the tricarboxylic acid cycle (TCA), upon further conversion to *α*-ketoglutarate in a reaction catalyzed by glutamate dehydrogenase (GDH). Exceeding substrates from the TCA cycle can be again available in the cytosol where they become the precursors of several anabolic pathways leading to biosynthesis of lipids, other aminoacids, and nucleotides. Beside its relevant role in anabolic pathways, glutamine metabolism may also promote further accumulation of lactate (via malate formation) and therefore exacerbate glycolysis and nicotinamide adenine dinucleotide phosphate-oxidase (NADPH) generation (glutaminolysis), the latter further buffering potential oxidative stress into the cells. Studies highlight that specific forms of cancer including glioblastoma develop an impressively high rate of glutamine metabolism, which goes beyond the real nitrogen demand, thus suggesting that glutamine consumption in cancer cells may represent a fast and preferential carbon source to replenish several biosynthetic pathways [30]. This preferential use of glutamine may be further promoted by other factors, whose expression level is altered in cancer cells, for example, the NFE2-related factor (NRF2) [31]. Altogether these observations imply that cancer cells may become addicted to glutamine metabolism to maintain their high rate of proliferation. Therefore, targeting their ability to degrade glutamine may be of therapeutic relevance especially in glutamine-dependent types of cancer.

#### 2.2.2. Lipid Metabolism

A growing body of evidence depicts a determinant role of altered lipid homeostasis in enabling the cancer cell phenotype. The pattern of alterations described suggests that lipid metabolism plays a multitasking role in cancer. Beyond the relevance of metabolic modifications that promote lipogenesis and therefore specific anabolic activities, lipid-related factors appear essential in controlling redox homeostasis and accumulation of specific lipid messengers, including lysophosphatidic acid and prostaglandins. Accordingly, several enzymes and transcription factors controlling lipogenesis and lipid homeostasis are overexpressed in cancer, as we will detail later. These alterations were initially identified in hormone-dependent malignancies such as those affecting breast [32] and prostate [33], thus confirming the relevance of steroid hormone-dependent pathways in the observed altered lipid metabolism. More recently, comparable patterns of alterations were identified in other cancer cell lines derived from melanoma [34], osteosarcoma [35], colorectal [36, 37], and lung cancer [38], as well as in hematopoietic cancer cells [39, 40]. These cellular environments allowed to identify additional modulatory upstream pathways including mitogen-activated protein kinase-(MAPK-) dependent [41], phosphatidylinositol-3-kinase (PI3K)/Akt pathway [41, 42], H-ras [41] and AMP-activated protein kinase, AMPK [43]. In addition, a lipid-related transcription factor, the sterol regulatory element-binding protein (SREBP), whose target genes promote cancer aggressiveness [44], is upregulated in cancer.

It is well-known that fatty acid neosynthesis is triggered by excess glucose leading to increased mitochondrial citrate concentrations. Citrate is then converted in the cytoplasm into palmitoyl-CoA, the precursor of triglycerides, and phospholipids synthesis. Accumulation of triglycerides may be reverted after starvation when a decrease of the lipogenic intermediate malonyl-CoA reactivates carnitine palmitoyltransferase-1 (CPT-1), thus leading to mitochondrial fatty acid oxidation [45]. 

In cancer cells, *de novo* fatty acid synthesis is sustained and several lipogenic enzymes are typically upregulated. The consequent burst in lipidogenesis confers the advantage to further exacerbate additional biosynthetic anabolic activities enabling cell growth. The enzymes ATP-citrate lyase (ACL), acetyl-CoA carboxylase (ACC), and the fatty acid synthase (FAS) are frequently overexpressed in cancer cells [46]. Especially FAS was described as a potential cancer biomarker [47, 48] for therapeutic purposes [49]. This dual clinical potential is supported by the observation that FAS inhibitors suppress carcinogenesis in *in vivo* procarcinogenic models of breast [50] and lung [38] tissues; moreover, they trigger cell death in a number of cancer cell lines [34, 47, 51–53], without affecting normal lipogenic tissues [54]. Additionally, FAS expression correlates with metastasis formation [35] and its targeting alleviates chemoresistance when combined with chemotherapeutic agents [55]. These multiple anticancer activities together with the observation that FAS are overexpressed in premalignant lesions [56, 57] strongly point at a very early role of FAS overexpression in carcinogenesis and led to the speculation that this enzyme may effectively be considered an oncogene [58–60].

Besides, cancer cells show a preferential synthesis of phospholipids (i.e., lysophosphatidic acid) instead of triglyceride [49]. This biosynthetic diversion of lipid precursors leads to the accumulation of lipid messengers regulating a number of signalling events promoting cancer cell growth, survival and migration to other tissues [61]. An accumulation of prostaglandins (i.e., prostaglandin E) strengthens the procarcinogenic roles played by proinflammatory signalling events during carcinogenesis [62]. Remarkably, a tight cross-talk exists between lipid metabolism and modulation of the expression of the main proinflammatory mediator cyclo-oxygenase 2 (COX-2), which is constitutively overexpressed in cancer [62, 63]. In line with these observations is also the fact that lipolytic enzymes like the monoacylglycerol lipase (MAGL) [64] are overexpressed in cancer and may directly control the prostaglandin levels [65]. 

Taking into account recent publications about the roles of lipid metabolism in cancer, we are convinced that further discoveries will further strengthen the importance of these pathways in cancer treatment and prevention.

### 2.3. Role of Altered Metabolism in Promoting Specific Cancer Hallmarks

Cell death resistance and angiogenesis are two important pathways involved in tumour progression and survival [66–68]. These independent processes are closely linked to cancer cell metabolism [67, 69]. Recent publications highlight mitochondria as modulators of these two critical pathways and promoters of metabolic homeostasis in cancer cells [70]. The mitochondrion is the most important coordinator of both energy production and accumulation of biosynthetic precursors for cellular maintenance and survival. 

Altered mitochondrial bioenergetics and functions play an important role in tumorigenesis by affecting cancer cell metabolism, decreasing mitochondria-dependent apoptosis, and contributing to angiogenesis [66, 70–72]. Cancer cells present frequently a mitochondrial metabolic shift from glucose oxidation (GO) to glycolysis, thus assimilating a larger amount of glucose compared to normal cells [73]. By this way, cancer cells refuel themselves with phosphorylated intermediates required for growth and proliferation, through regulation of the metabolic key enzymes that govern the balance between GO to glycolysis and by reducing the entry of pyruvate into mitochondria thus reducing the rate of TCA cycle [17, 73, 74]. The accumulated pyruvate is in part converted to lactate during aerobic glycolysis and secreted to keep glycolysis active. The extracellular secreted lactate influences the extracellular matrix lowering the pH of the tumour environment, allowing a remodelling of the matrix and inducing blood vessel invasion in response to tumour-induced angiogenic factors [17]. Therefore, the reduced mitochondrial efficiency may induce the activation of HIF-1*α* resulting in angiogenesis activation, cell migration, increased cell survival, and energy metabolism [75, 76]. Conversely, restoration of the mitochondrial activity inhibits HIF-1*α* [77–79]. It has been demonstrated that dichloroacetate (DCA), which inhibits pyruvate dehydrogenase kinase (PDK), activates GO in mitochondria thus leading to decreased tumour growth in many cancer cell lines; this event is accompanied by the inhibition of HIF-1*α* [69].

Alterations in mitochondrial function not only influence the cellular metabolic status but also contribute to the control of the redox status of cancer cells. The large amounts of glucose available in the cells are metabolized through the pentose phosphate pathway (PPP) producing nucleosides and generating NADPH [70, 73].

NADPH is essentially involved in redox control protecting cells against ROS. High levels of ROS, as generated in cancer cells, can promote oxidative damage-induced cell death. Therefore, cancer cells maximize their ability to produce NADPH to reduce ROS activity [73]. The difference in the redox status between normal and cancer cells may be a target to selectively kill cancer cells by ROS-generating drugs. Thus, the elicitation of ROS can be exploited to induce cancer cells to undergo oxidative damage-induced cell death.

Another important modulator of the redox status in cancer cells is B-cell lymphoma-2 (Bcl-2) protein that is, overexpressed in a variety of cancer cells [80]. The potential tumorigenic activity of Bcl-2 is due to its antiapoptotic properties maintaining the integrity of the outer mitochondrial membrane and preventing its permeabilisation through sequestration of the proapoptotic protein B-Cell lymphoma-associated X (BAX) and Bcl-2 homologous antagonist killer (BAK). However, regulation of ROS levels by Bc-2 was also demonstrated [81, 82] as Bcl-2 may affect the intracellular redox status in order to maintain the ROS potential at the most favorable level for cancer cell survival.

Autophagy is another alternative pathway that sustains tumour cell survival. Moreover, autophagy is a major process fueling cell metabolism [67]. It supplies intracellular nutrients when the external ones are not available. Unlike normal cells, cancer cells are placed in an environment deprived of nutrients and oxygen due to an insufficient vascularization. Autophagy may support tumour growth ensuring the availability of endogenous metabolic substrates necessary to feed glycolysis, ATP production, and pyruvate for the mitochondrial metabolism [67]. Autophagy recycles intracellular organelles and the resulting breakdown products contribute to produce energy and build up new proteins and membranes. Indeed, autophagy provides an internal source of sugar, nucleosides, amino acids, and fatty acids by the degradation of protein, lipids, carbohydrate, and nucleic acids [83]. Thus, autophagy sustains cell metabolism and subsequently favors cancer cell survival in nutrient lacking tumours, besides preventing that cancer cells may accumulate dysfunctions in their mitochondria [84].

Impaired mitochondrial functions, oxidative stress, and autophagy are tightly correlated. Emerging evidence underlines how much autophagy may affect mitochondrial functions and accumulation of ROS [85]. Number and the health status of mitochondria are controlled by an autophagic process called mitophagy. Mitophagy is a mitochondrial quality control by means of which excessively damaged mitochondria become a substrate for autophagic degradation. Hypoxia and hypoxia-inducible factors (HIFs) can induce mitophagy [86]. Dysfunctional mitochondria are linked to ROS generation, induction of DNA damage, and cell death [87]. Thus, degradation of these defective organelles by mitophagy may protect cells from carcinogenesis. However, both activation and inhibition of the autophagic pathways may play a role in cancer therapy. It has been demonstrated that inhibitors of autophagy may target autophagy-dependent cancer cells because this modulation inevitably impairs cancer cell survival [88]. On the other side, an excessive autophagic flux can induce cell death. Therefore, cytotoxic cancer therapies exacerbating autophagy may provoke increased oxidative stress or severe cell damage, thus sensitizing cancer cells to cell death (i.e., apoptosis) [89]. Interestingly, autophagy and apoptosis are both regulated by Bcl-2. Bcl-2 regulates autophagy by binding to the proautophagy protein Beclin-1 and the proapoptotic protein Bax [72]. Therefore, the cross-talk between autophagy and the mitochondrial metabolism is an important issue to be considered for cancer therapy. Moreover, redox alterations associated with mitochondrial dysfunctions may be pivotal in preventing cancer formation, growth, and establishment at very early steps of carcinogenesis.

## 3. Potentially Targetable Metabolic Actors by Natural Compounds

The logical consequence of the elucidation of the multiple roles played by altered metabolism in cancer is the exploitation of this knowledge for preventive and therapeutic purposes. The existence of specific patterns of modulations identifies also potential molecular targets for future novel classes of anticancer compounds. In this section, we suggest an overview of natural compounds regulating the most interesting metabolic pathway intermediates.

### 3.1. Glycolysis-Related Factors

#### 3.1.1. Glucose Transporters

It is essential for a cancer cell to activate the glycolytic pathway to satisfy the anabolic demand in consistent amounts of intracellular glucose. Glucose is carried into cells via specific plasma membrane transporters that lead to glucose internalization by facilitation or active coupling to ion fluxes like the extrusion of Na^+^ [90]. 

**Figure 1:**
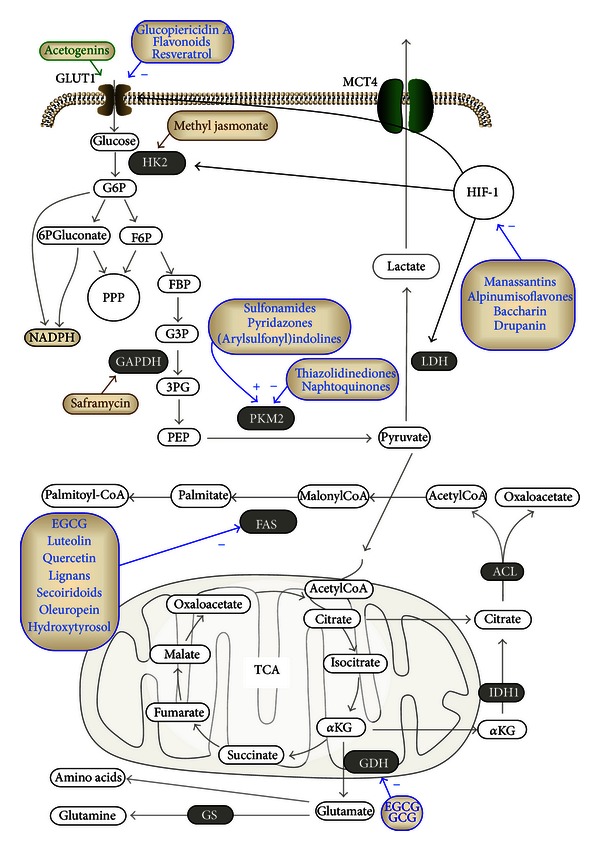
Targetable metabolic actors by natural compounds. A summary of the most relevant compounds affecting metabolic pathways of cancer cells. Many of these molecules correspond to natural compounds; alternatively, they are chemical structures found as active that may act as a template for the identification of promising natural compounds with similar activity. Molecules indicated in blue affect enzymatic activity (+ or − stands for activators or inhibitors, resp.); the ones in green affect the expression level of the targeted enzyme; the ones in brown affect nonmetabolic activities. Abbreviations: ATP citrate lyase, ACL; gallocatechin gallate, GCG; epigallocatechin gallate (EGCG); fatty acid synthase, FAS; fructose-6-phosphate, F6P; fructose-1,6-biphosphate, FBP; hypoxia-inducible factor 1, HIF-1; glucose-6-phosphate, G6P; glutamine synthetase, GS; hexokinase II, HK2; glyceraldehyde-3-phosphate, G3P; glyceraldehyde-3-phosphate dehydrogenase, GAPDH; glucose transporter, GLUT; glutamate dehydrogenase, GDH; *α*-ketoglutarate (*α*KG); isocitrate dehydrogenase 1, IDH1; lactate dehydrogenase, LDH; nicotinamide adenine dinucleotide phosphate-oxidase, NADPH; pentose phosphate pathway; 3-phosphoglycerate, 3PG; phosphoenolpyruvate, PEP; pyruvate kinase isoform M2, PKM2.

Frequently, specific isoforms of glucose transporters are overexpressed in cancer cells. The facilitative glucose transporters (GLUTs) belonging to the solute carrier (SLC2) gene family are frequently overexpressed. Consistent data was published about isoforms 1, 3, 4, and 12. Therefore, targeting abnormal expression or activity of those carriers represents one promising strategy. Several natural compounds have been described as potential modulators of glucose transporters (Figure 1). A critical reading of the literature indicates that these compounds most likely affect expression of glucose transporters indirectly, rather controlling upstream modulatory mechanisms. This is also true for natural compounds. Annonaceous acetogenins are long chained fatty acid derivatives extracted from different tropical plants such as the tree *Annona muricata*, also known as Graviola. It has been recently shown that Graviola extracts exert multiple anticancer activities on pancreatic cancer cell models [91]. The extract reduces cell proliferation and viability by inducing necrosis; besides, it counteracts cell motility. The potential anticancer properties have been confirmed with mouse xenograft models, where Graviola extract reduces both tumour growth and formation of metastasis. An analysis centered on metabolic parameters underlines the ability of this compound to inhibit glucose uptake; besides, it strongly reduces the expression levels of several metabolic actors, including GLUT1 and GLUT4, HKII, and LDH-A. This pattern of modulation is the consequence of the modulation of multiple factors and pathways including the reduction of HIF-1 and nuclear factor *κ*B (NF-*κ*B) expression levels and the inhibition of ERK (extracellular-regulated kinase) and Akt activation. 

Due to the difficulties of specifically targeting glucose transporter expression without affecting many other intracellular pathways, an interesting alternative is to identify molecules that modulate the activity of glucose transporters. In this context several natural compounds deserve attention.

Following a natural product screening assay based on crude extracts of microbial origin aimed at identifying new inhibitors of filopodia protrusion (special membrane structures involved in promoting metastasis), Kitagawa and colleagues have isolated and characterized in the broth of *Lechevalieria sp.* bacterial strain glucopiericidin A (GPA) as a novel inhibitor of glycolysis [92]. The authors showed that GPA specifically impairs glucose uptake into the cells. Accordingly, the compound impairs the accumulation of the nonmetabolizable tritiated glucose analog 2-deoxyglucose (DG) without affecting the key glycolytic enzyme HK. Their findings suggest that GPA may act by mimicking a GLUT1 substrate. 

From plants, polyphenols are interesting bioactive anticancer molecules as several of them have been repeatedly reported to control glucose transporter activity in different cancer cell models; fisetin, myricetin, quercetin, apigenin, genistein, cyaniding, daidzein, hesperetin, naringenin, and catechin are well-known inhibitors of glucose uptake [93]. Investigations designated hexose and dehydroascorbic acid transporters including GLUT1 and GLUT4 [94, 95] as their targets. Comparative studies indicate that these compounds do not exhibit the same mode of action as they bind different domains of GLUT1. Genistein binds the transporter on the external face whereas quercetin interacts with the internal face [95]. The ability of these compounds to act as protein-tyrosine kinase inhibitors is currently considered as the main mechanism responsible for the modulation of the glucose uptake.

#### 3.1.2. Glycolytic Enzymes

Hexokinase (HK) is the enzyme controlling the first enzymatic step of glycolysis, allowing intracellular transformation of glucose via phosphorylation (Figure 1). In cancer cells, HKII is the main isoform and is involved in the Warburg effect and in enhanced cell proliferation [96]. HK associates with the outer mitochondrial membrane in proximity of ATP molecules required for HK's enzymatic activity. The destabilization of this physical interaction negatively affects the overall cancer cell energetics; moreover, it dramatically perturbs mitochondria, triggering the release of cytochrome c and, subsequently, inducing apoptosis [97]. Some natural compounds have been described as promoting the detachment of HK from mitochondria. Methyl jasmonate is a plant stress hormone produced by many plants including rosemary (*Rosmarinus officinalis L.*), olive (*Olea europea L.*), or ginger (*Zingiber officinalis*); it binds to HK and perturbs its association with the voltage-dependent anion channel (VDAC) in cancer cells [98]. This event leads to overall energetic impairment; moreover, it promotes the release of cytochrome c from mitochondria, triggering apoptosis. Its use in combination with the antiglycolytic agent 2-deoxyglucose or chemotherapeutic agents is currently under investigation [98].

Glyceraldehyde-3-phosphate dehydrogenase (GAPDH) is a key glycolytic enzyme catalyzing the conversion of glyceraldehyde-3-phosphate to glycerate 1,3-biphosphate, accompanied by the generation of NADH. There is evidence that GAPDH may play multiple noncanonical functions implicated in cell growth and survival. The *bis*-quinone alkaloid saframycin, a bacterial product of fermentation, exhibits antiproliferative properties in both adherent and nonadherent cancer cell models. This compound possesses activities comparable to alkylating agents. The group of Myers has shown that saframycin may form a nuclear ternary complex with GAPDH and DNA [99] involved in the antiproliferative effect ascribed to this compound [99]. 

Recently, the embryonic isoform M2 (PKM2) is attracting interest for diagnostic and therapeutic purposes in cancer [5]. Enzymes of the pyruvate kinase family catalyze the final, rate-limiting, step of glycolysis, leading to the accumulation of pyruvate from phosphoenolpyruvate in an ATP-producing reaction. Cancer cells exclusively express the embryonic isoform M2 instead of adult M1. This switch is required for the maintenance of aerobic glycolysis [8]. Also rapidly proliferating cells selectively express PKM2. Importantly, PKM2 exists as a dimeric or a tetrameric form; the latter one efficiently catalyzes pyruvate formation whereas the dimeric form is nearly inactive. In cancer cells the dimeric form is the preponderant one. This paradoxical behavior is believed to further promote glycolysis and several anabolic activities. 

Currently two main PKM2 targeting strategies are under evaluation. The first attempt consists in identifying compounds inhibiting PKM2. High-throughput screenings based on an enzymatic LDH assay to explore a compound library including molecules approved from the Food and Drug administration (FDA) and purified natural products have led to the identification of three potential chemical structures associated with a potential inhibitory activities on PKM2 [100]. Active compounds include thiazolidinediones and natural compounds belonging to the group of naphthoquinones: shikonin, alkannin, and their derivatives (extracted from different plants including *Arnebia sp*. and *Alkanna tinctoria*) have been recently shown as the most potent and specific inhibitors of PKM2 [101]. These compounds reducing lactate production and glucose consumption in cancer cells are also known to induce necroptosis [102]. However, the inhibitory effect on PKM2 is independent of their effect on cell viability, rather suggesting an impairment of the glycolytic metabolism. Even though PKM2 is crucial for cancer cell survival [101], there is a potential risk to affect also healthy PKM2-expressing cells. Subsequently, a second line of research currently aims at promoting the reactivation of PKM2 in cancer cells. The increase of tetrameric versus dimeric PKM2 isoform ratio abrogates the Warburg effect and may reactivate oxidative phosphorylation [103]. So far a few promising studies have been published identifying some chemical scaffolds as potential PKM2 activators. They include sulfonamides, thieno[3,2-b]pyrrole[3,2-d]pyridazinones, and 1-(sulphonyl)-5-(arylsulfonyl)indolines that act as small-molecule allosteric modulators binding to a surface pocket of the enzyme, thus facilitating the association of different PKM2 subunits.

Although PKM2 targeting appears a promising area for drug discovery, research remains preliminary. Identification of first chemical scaffolds may be the basis for the discovery of structurally related natural compounds.

### 3.2. Hypoxia-Inducible Factor-1: The Hypoxic Rheostat

There is no doubt that HIF-1 is a central molecule in the control of the expression of glucose transporters and key glycolytic enzymes as well (Figure 1). Accordingly, an important strategy is the identification of small molecule inhibitors of HIF-1. Several attempts rely on cell-based assays with reporter gene constructs under the control of a HIF-1 response element. The group of Zhou has discovered and characterized novel HIF-1 inhibitors in (i) manassantins (manassantin B and 4-o-demethylmanassantin) extracted from the aquatic plant *Saururus cernuus* [104] and (ii) alpinumisoflavones (alpinumisoflavone and 4′-O-methyl alpinumisoflavone) isolated from the tropical legomaceous plant *Lonchocarpus glabrescens *[105]. These compounds inhibit hypoxia-induced HIF-1 activation; besides, they may affect the expression of HIF-1 and HIF-1 target genes including GLUT1 and/or VEGF. Similarly, the group of Nagasawa has identified the cinnamic acid derivatives baccharin and drupanin, extracted from the Brazilian green propolis as inhibitors of HIF-1-dependent luciferase activity [106]. They inhibit the expression of HIF-1 and its target genes (GLUT1, HKII, and VEGF); besides, they exhibit antiangiogenic effects.

### 3.3. Modulation of Mitochondrial Metabolism and Functions

Several natural compounds have been shown to be able to target mitochondrial metabolism and functions, besides affecting cell death and angiogenesis, both important pathways involved in cancer progression.

Curcumin is a natural compound extracted from *Curcuma longa*, widely used as a spice. Its anticarcinogenic and chemopreventive effects target mitochondrial metabolism and function inducing cell death and angiogenesis in a variety of cancer models [107]. In human colorectal carcinoma cells, curcumin induces mitochondrial membrane potential, induces procaspase-3 and -9 cleavage and apoptosis in a dose- and time-dependent manner accompanied by changes, and release of lactate dehydrogenase. It leads to cell cycle arrest in S phase, accompanied by the release of cytochrome c, a significant increase of Bax and p53 levels, and a marked reduction of Bcl-2 and survivin in LoVo cells [108].

Dimethoxycurcumin (Dimc), a synthetic analogue of curcumin, induces cell cycle arrest in S phase and apoptosis in human breast carcinoma MCF-7 cells by affecting mitochondrial dysfunction by oxidative stress. Accordingly, it was observed that DNA damage and apoptosis followed an induction of ROS generation and a reduction of glutathione levels [109]. Mitochondrial dysfunction was also witnessed by a reduction of the mitochondrial membrane potential and a decrease of the cellular energy status (ATP/ADP) by the inhibition of ATP synthase. Therefore, the mitochondrial dysfunctions correlated with changes in the expression of apoptotic markers like Bax and Bcl-2 [109]. Several studies indicated redox alterations as a causative mechanism implicated in mitochondrial dysfunction in cancer. Chen et al. published a novel pathway for curcumin regulation of the ROS-lysosomal-mitochondrial pathway (LMP) and identified cathepsin B (cath B) and cathepsin D (cath D) as key mediators of this pathway in apoptosis. In lung A549 cancer cells, curcumin induces apoptosis via lysosomal membrane permeabilisation depending on ROS increase, which precedes the occurrence of mitochondrial alterations [110]. Further studies demonstrated that curcumin-induced ROS generation decreases the mitochondrial membrane potential followed by downregulation of Bcl-2 expression, Bax activation, and release of cytochrome c into the cytosol, paralleled by the activation of caspase-9 and -3 in small cell lung cancer (SCLC) and NPC-TW 076 human nasopharyngeal carcinoma cells [111, 112].

Curcumin-induced apoptosis in the colon cancer cell line HCT116 is significantly enhanced by the suppression of mitochondrial NADP(+)-dependent isocitrate dehydrogenase activity which plays an essential role in the cell defense against oxidative stress by supplying NADPH for the antioxidant systems [113].

Amaryllidaceae alkaloid pancratistatin isolated from the bulb of *Hymenocallis littoralis* exhibits potent apoptotic activity against a broad panel of cancer cells lines with modest effects on noncancerous cell lines [114]. Pancratistatin led to ROS generation and mitochondrial depolarization, leading to caspase-independent cell death in breast carcinoma cells. In colorectal carcinoma cell lines, but not in noncancerous colon fibroblast cells, pancratistatin decreased mitochondrial membrane potential and induced apoptotic nuclear morphology independently on Bax and caspase activation [114]. In colon cancer cells, resveratrol, a natural stilbene from grapes, blueberries, or cranberries, induces apoptosis by nitric oxide production and caspase activation [115]. Conversely, in multiple myeloma cells resveratrol increased apoptosis, by blocking the activation of NF-*κ*B and subsequently downregulation of target genes including interleukin-2 and Bcl-2, leading to cell cycle arrest [116].

The cross-talk between mitochondria and the autophagic machinery could be used as a therapeutic strategy. Resveratrol has several beneficial effects such as neuroprotection and cytotoxicity in glioblastoma cell lines. It has been demonstrated that resveratrol induced a crosstalk among autophagy and apoptosis to reduce glioma growth [117]. Indeed, resveratrol has an impact on the formation of autophagosomes in three human GBM cell lines, accompanied by an upregulation of autophagic proteins Atg5, beclin-1 and LC3-II [117]. However, the inhibition of resveratrol-induced autophagy triggered apoptosis with an increase in Bax expression and cleavage of caspase-3. Only the inhibition of both cell death pathways abrogated the toxicity of resveratrol. Thus, resveratrol activates autophagy by inflicting oxidative stress or cell damage, in order to sensitize glioblastoma cancer cells to apoptosis [117]. Also, curcumin treatment of human liver-derived HepG2 cells induces the reduction of mitochondrial membrane potential and the activation of autophagy. Moreover, it has been demonstrated that curcumin activates mitophagy. This finding underlines the importance of mitophagy in the process of cell death of nasopharyngeal carcinoma cells [118]. 

As mentioned earlier, another important pathway in mitochondrial dysfunction involved in tumour progression is HIF-1*α*. It has been published that curcumin plays a pivotal role in tumour suppression via the inhibition of HIF-1*α*-mediated angiogenesis in MCF-7 breast cancer cells and in HepG2 hepatocellular carcinoma cells [119, 120]. Anticancer activity of curcumin is attributable to HIF-1 inactivation by Aryl hydrocarbon nuclear translocator (ARNT) degradation. Another natural compound with a potent antiangiogenic activity is the flavonoid bavachinin. Bavachinin inhibited increased HIF-1*α* activity in human KB carcinoma derived from HeLa cells [121]. In human HOS osteosarcoma cells under hypoxia, bavachinin decreased transcription of genes associated with angiogenesis and energy metabolism that are regulated by HIF-1, such as vascular endothelial growth factors (VEGFs), GLUT1, and HKII [121]. Bavachinin may be used as a therapeutic agent to inhibit tumour angiogenesis. Indeed, *in vivo* studies showed that injecting bavachinin significantly reduced tumour volume in nude mice with KB xenografts [121].

**Figure 2:**
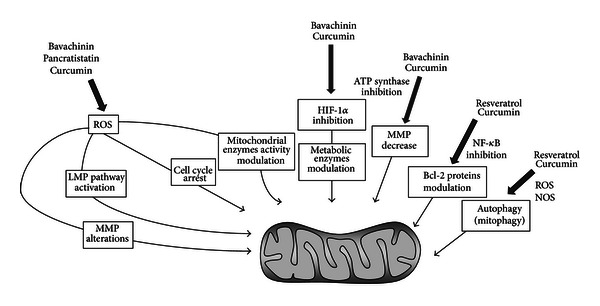
Mitochondrial dysfunctions as pharmacological targets. Examples of natural compounds with a potential efficacy in cancer treatment. The figure schematizes their mechanism of action linked to mitochondrial dysfunctions. The compounds discussed herein have different mitochondrial targets, such as mitochondrial membrane potential (MMP), Bcl-2 family proteins (Bcl-2), reactive oxygen species (ROS), HIF-1*α*, mitochondrial metabolism (MM), and autophagy.

Figure 2 summarizes the major mechanisms of action described for natural compounds as mitochondrial modulators.

### 3.4. Targeting Other Altered Metabolic Pathways in Cancer Cells

#### 3.4.1. Glutamine Metabolism

Glutamine and glucose are the main carbon sources used by cancer cells to satisfy their anabolic demand. Published data indicate a role for glutamine metabolism within the malignant cell phenotype. Accordingly, several cancer cell lines present a high rate of glutamine consumption and strategies are investigated to target enzymes implicated in this pathway. Inhibiting the activity of glutamate dehydrogenase (GDH) is an effective anticancer strategy as documented in glioblastoma cells with combinatorial treatments with agents depleting cells of glucose or inhibiting specific kinase-(i.e., AKT-) dependent pathways [122]. Polyphenols extracted from green tea including epigallocatechin gallate (EGCG) and catechin gallate (CG) inhibit GDH, by recognizing and binding to the site of the allosteric regulator ADP [123, 124]. These findings allow to speculate about the potential use of these polyphenols and of their derivatives with improved bioavailability in the treatment of glutamine-dependent forms of cancer.

#### 3.4.2. Lipid Metabolism

FAS sustains the altered lipid metabolism in cancer cells. As discussed in Section 2.2.2, several reports support the relevance of this enzyme as a target in cancer cells. This enzyme is a complex system with seven different functional domains [125]. This property amplifies the possibility of impairing its enzymatic activity with different specific compounds. 

Four major specific FAS inhibitors are known [126]. The antibiotic cerulenin (extracted from the fungus *Cephalosporium caerulens*) acts as noncompetitive inhibitor of the *β*-ketoacyl synthase domain [127]. Tetrahydrolipstatin, also known as Orlistat (a derivative of the natural compound lipstatin), targets the thioesterase domain of FAS [128]. Triclosan affects the enoyl-reductase activity of the enzyme [129]. Finally, the synthetic chemical derivative of cerulenin C75 is the most potent compound *in vitro* able to affect all the three domains mentioned earlier in a competitive irreversible way [129]. Orlistat was approved by the Food and Drug Administration (FDA) for its ability to reduce body weight. Besides, all these molecules display anticancer activities by blocking cancer cell proliferation and triggering cancer cell death [126]. Nevertheless, their actual application for cancer treatment is hindered by several side effects, which include their ability to modulate other enzymes (i.e., the increase of CPT-1 activity and fatty acid oxidation by cerulenin and C75 leading to weight loss [130, 131]), their reduced bioavailability (i.e., Orlistat [126]), or stability *in vivo* (i.e., C75 inactivation by intracellular glutathione and other small thiols [132]). Current research efforts focus on the design of new synthetic derivatives of this first group of molecules, on one side, and on the identification of new compounds of natural origin, on the other side, both potentially showing improved characteristics of specificity and bioavailability/stability *in vivo*. 

In this context, the potential identification of new FAS inhibitor from natural compounds is a particularly interesting strategy, especially by investigating compounds of vegetal origin showing the double favorable profile of being regularly consumed in the diet and displaying at the same time hypolipidemic and anticancer activities. Several classes of polyphenols appear as very good candidates. Extracts from green and black tea have been repeatedly proved as lipidogenic inhibitors [133]. Further investigations have identified catechin gallate derivatives (including EGCG, epicatechin gallate (ECG), and catechin gallate (CG)) as specific FAS inhibitors as demonstrated by *in vitro* assays of FAS enzymatic activity [134, 135]. The galloyl moiety of the catechins is essential for the inhibitory activity of these molecules; it directly interacts and modulates the function of the *β*-ketoacyl reductase domain of FAS [134, 135]. The FAS inhibitory activity is common to other polyphenolic compounds. The group of Tian has first described several flavones including luteolin, quercetin, kaempferol, myricetin, fisetin, and baicalein as inhibitors of the *β*-ketoacyl reductase domain [136]. The flavone luteolin and the flavonols quercetin and kaempferol (and with a lower extent the flavone apigenin and the flavanone taxifolin) have been shown to act as potent inhibitors of lipogenesis in a comparative study with EGCG in prostate cancer [137]. An *in vitro* FAS enzymatic activity assay confirmed their ability to inhibit FAS, however, less potently compared to ECGC [137]. Tian and colleagues suggested that all polyphenolic FAS inhibitors share a biphenyl core potentially responsible for their described inhibitory activity [138]. Possible differences may account for a structure-dependent mechanism of action, where flavones as quercetin and kaempferol containing hydroxyl groups at specific positions [137] display a reversible fast binding inhibitory activity, whereas EGCG and ECG exhibit an irreversible slow binding activity [134]. It has been taken into account, however, that further variability may be associated with differential uptake, metabolization, and intrinsic stability of the compounds. Finally, the effects on lipid metabolism may be the result of multiple intracellular signalling events, modulated by polyphenolic compounds and eventually converging towards the control of the lipid metabolism. Curcumin has been shown to affect lipid accumulation and FAS activity [139]. This ability may be partially linked to the known antagonistic activity of this compound towards the NF-*κ*B-mediated pathway [140]. Besides, curcumin and its derivatives have recently been shown to modulate the AMPK-SREBP pathway [141, 142]. Green tea extracts prevent EGF-induced upregulation of FAS in MCF-7 via modulation of a PI3 K/AKT-dependent pathway [143]. Other polyphenolic compounds have been identified as inhibitors of lipidogenesis by targeting FAS and/or the transcription factor SREBP expression through the modulation of specific pathways. These findings may therefore suggest further relevant pathways involved in the control of the lipid metabolism in cancer cells. For example, resveratrol, a stilbene contained in grapes, produces hypolipidemic effects by activating the NAD-dependent deacetylase sirtuin 1 (SIRT-1), which positively modulates AMPK [144]; AMPK activation, in turn, prevents lipid accumulation by controlling several events, including FAS downregulation [144]. The activation of AMPK by resveratrol has been also confirmed in other studies [145]. Moreover, it is a common property shared with compounds from other plants showing hypolipidemic properties, as observed with extracts from *Hibiscus sabdariffa* [146]. Promising interesting therapeutic implications may derive also from phenolic compounds contained in the extra-virgin olive oil, which was described as a very active inhibitor of FAS expression and controller of lipid biosynthesis in breast cancer cell models [147]. Compounds belonging to lignans (1-[+]-pinoresinol and 1-[+]-acetoxy-pinoresinol), flavonoids (apigenin and luteolin), and secoiridoids (deacetoxyoleuropein aglycone, ligstroside aglycone, oleuropein glycoside, and oleuropein aglycone) appear as the most active compounds, by activating AMPK and reducing SREBP-1 expression [147]. Similarly, polyphenols oleuropein and hydroxytyrosol from extra-virgin olive oil were able to inhibit FAS activity in colorectal cancer SW260 cells, and this effect correlated with their antiproliferative potential [148]. However, this effect could not be confirmed in another colon model (HT-29) suggesting cell-type specific effect and further unrelated mechanisms [148]. Targeting lipid metabolism and especially FAS activity remains a promising perspective to target cancer cell survival. Brusselmans and colleagues showed that palmitate added to the culture medium of prostate cancer cells allowed to bypass the downstream effects of FAS inhibition by luteolin on lipid metabolism and prevented the cytotoxic effect of this compound [137]; moreover, the silencing of FAS expression with FAS siRNA produced similar cellular alterations as luteolin [137]. These findings allow predicting a causative role of FAS inhibition in the antiproliferative and cytotoxic effect of polyphenols and prompt to explore the relevance of the control of the lipid metabolism by polyphenols in the anticancer activities ascribed to many of these compounds.

## 4. Concluding Remarks

The targeting of altered cell metabolism in cancer cell is a promising still unexplored area in anticancer strategies. In this review, we have highlighted that many of these modifications take place at very early steps of carcinogenesis, thus at preneoplastic stages. Therefore, their targeting may be a powerful weapon for chemopreventive purposes. Besides, the literature clearly shows the crucial addiction of cancer cells to several metabolic aberrations to proliferate and survive, further underlining the importance of the targeting of some metabolic-related factors in future anticancer therapies.

Identification of specific aberrantly regulated metabolic keynodes, in terms of the expression and/or activity of these factors, delineates the nature of potential pursuable molecular targets. Despite all these considerations, the effective number of agents under investigations for antimetabolic purposes is still very poor and at a very preliminary stage. Good candidates should present a favorable profile ensuring excellent differential toxicity against cancer versus healthy cells, a reduced risk of systemic toxicity, combined to a favorable profile in specific pharmacological properties including bioavailability, half-life, and stability. Many natural compounds have so far been identified as anticancer agents by affecting almost each cancer hallmark [2]. Taking into account recently identified cross-talks between altered metabolic mediators and altered proliferation, survival, or migration properties, we may suspect that many of the anticancer properties so far ascribed to natural compounds are mainly due to a their potential in modulating cellular metabolism. Remarkably, we have reported here many examples of dietary polyphenolic compounds from fruits and vegetables, which display specific antimetabolic functions (Figure 1). Although there is consistent evidence of multiple beneficial biological properties on health, there are yet some obstacles which hinder promising natural compounds from being already used for chemopreventive and therapeutic purposes including bioavailability and adsorption. Moreover, information concerning the stability and the clearance of natural occurring compounds frequently remains to be yet determined; additional efforts will be required towards the elucidation of this important properties in the next future. 

Nature represents an impressively huge “database” of different and diversified molecular scaffolds. Rapid advancement in new screening systems allowing the analysis of large libraries of isolated naturally occurring compounds offers new important and fast tools for the selection of promising antimetabolic compounds especially from dietary origins with reduced side effects. Relatively low costs for their extraction/production in large amounts make them interesting for commercial objectives and represent a good basis for chemical modifications that may further improve their anticancer activities and facilitate their pharmacological use and efficiency. 

## Abbreviations

AMPK: AMP-activated protein kinase
Atg5: Autophagy protein 5
ARNT: Aryl hydrocarbon nuclear translocator
Bcl-2: B Cell Lymphoma-2
BAX: B Cell Lymphoma-Associated X
BAK: Bcl-2 homologous antagonist killer
CO_2_: Carbon dioxide
CG: Catechin gallate
COX-2: Cyclo-oxygenase-2
DCA: Dichloroacetate
ENO-1: Enolase-1
ERK: Extracellular-regulated kinase
FAS: Fatty acid synthase
HIF: Hypoxia-inducible factor
EGCG: Epigallocatechin gallate
GCG: Gallocatechin gallate
HKII: Hexokinase II
HDACs: Histone deacetylases
GAPDH: Glyceraldehyde-3-phosphate dehydrogenase
GO: Glucose oxidation
GLUT: Glucose transporter
GDH: Glutamate dehydrogenase
LDH: Lactate dehydrogenase
LMP: Lysosomal-mitochondrial pathway
MAPK: Mitogen-activated protein kinase
MMP: Mitochondrial membrane potential
NAD: Nicotinamide adenosine diphosphate
NF-*κ*B: Nuclear factor *κ*B
NADPH: Nicotinamide adenine dinucleotide phosphate-oxidase
PPP: Pentose phosphate pathway
PI3K: Phosphatidylinositol-3-kinase
PDK: Pyruvate dehydrogenase kinase
PKM2: Pyruvate kinase isoform M2
ROS: Reactive oxygen species
STAT3: Signal Transducer and Activator of Transcription 3
SREBP: Sterol regulatory element-binding proteins
TCA: Tricarboxylic acid cycle
VEGF: Vascular endothelial growth factors.
